# Starting from Scratch: Experiences from Developing the First Vascular Anastomotic Training Program in North Macedonia Using the Porcine Thigh as a Simulation Model

**DOI:** 10.1055/s-0044-1779474

**Published:** 2024-04-04

**Authors:** Eleonora O.F. Dimovska, Gordana Georgieva, Blagoja Srbov, Boro Dzonov, Goran Stevanovski, Sofija Pejkova

**Affiliations:** 1 Plastic and Maxillofacial Department, Uppsala University Hospital, Uppsala, Sweden; 2 Department of Plastic and Reconstructive Surgery, University Clinic Skopje, North Macedonia; 3 Faculty of Medicine, University Ss. Cyril and Methodius, Skopje, North Macedonia

**Keywords:** microsurgery course, plastic surgery training, vascular anastomosis

## Abstract

Microsurgical reconstruction constitutes a fundamental part of plastic and reconstructive surgery. It demands high dexterity and intricate technical skills. Its steep learning curve benefits from thorough training throughout residency, where using realistic simulation models in the appropriate sequence of complexity progression is essential in ensuring patient safety prior to progressing to a clinical setting. Commencing training on microvascular-like small diameter vessels could prove unsuitable and ineffective for inexperienced surgeons, however, the larger diameter neurovascular structures in the porcine thigh can provide excellent anastomotic training without compromising the animal tissue training sought after by residents. We present the results from implementing the first vascular anastomotic course in our country, where reconstructive theory was combined with simulated anastomotic training on the porcine thigh. Junior plastic surgery residents described acquiring comprehensive knowledge of reconstructive techniques and could successfully complete anastomoses, despite none to minimal previous experience. Using the porcine thigh should be encouraged as a start-up vascular anastomotic training tool as it provides realistic conditions and tissue handling training, and could improve quality of further training on microvascular structures.

## Course Method and Simulation Porcine Model

**Fig. 1:**
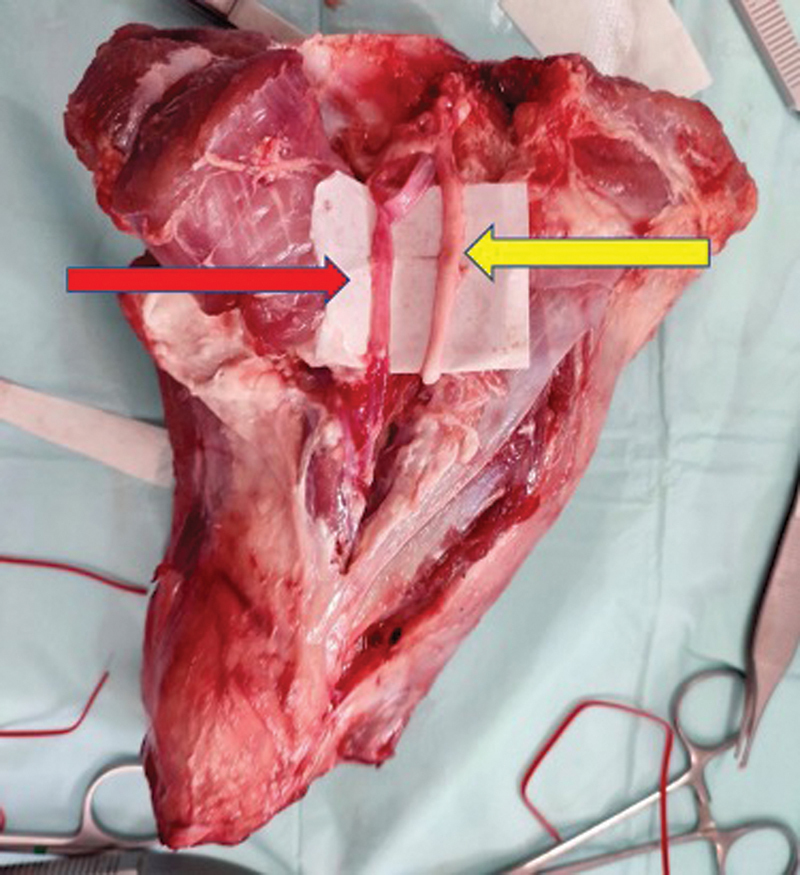
Exposure of the femoral artery (red arrow) and tibial nerve (yellow arrow) between the vastus medialis muscle and the sartorius above the knee, of the porcine thigh model.

In October 2022, a reconstructive microsurgery course was held over 4 days, offering both theoretical and hands-on practical training. Sixteen first-to-final year residents participated, with most having minimal to no prior microsurgical experience. Only one had previously executed a microsurgical anastomosis in an actual clinical environment. The faculty team comprised the authors, local plastic surgery consultants, and a medical education specialist. In the quest for finding the most effective simulation model, both chicken and porcine thighs were evaluated, and the latter was chosen due to its large vessel and nerve sizes (about 5 mm diameter), considered ideal for novices. The porcine thigh was dissected as described by Nam et al. 1 A linear incision was made on the fascia extending down to the pedicle. The femoral vessels were found between the vastus medialis muscle and the sartorius above the knee carefully dissecting the intermuscular septum between these muscles ( Fig. 1 ). All porcine thighs were partially prepared for our residents and the course efficacy was evaluated via an 18-question Likert scale questionnaire ( Table 1 ).

**Table 1:** Eighteen-question 5-point Likert scale questionnaire

1.	I will use the skills learned in the course in the future
2.	The lectures will benefit my education
3.	Working in pairs had a positive effect
4.	The theoretical lectures adequately prepared me for the activities of the practical part of the course
5.	I am satisfied with the lectures and the workshop in general
6.	The level of the course was understandable for my level of education
7.	The educators helped me to understand the contents of the course
8.	The topic of the course was interesting to me
9.	I am satisfied with the level of interactivity of the course
10.	I would attend the next microsurgery course in our country
11.	The course motivated me to continue my education in the field of microsurgery
12.	I am satisfied with my contribution to the microsurgery course
13.	I would rather attend a microsurgery course in our country than abroad
14.	The duration of the course was enough to complete the given tasks
15.	The instruments were adequate to perform the task
16.	The present material helped me prepare for the assignment
17.	The choice of lecturers/educators on the course was appropriate
18.	I am satisfied with the overall organization of the event

Eighteen-question 5-point Likert scale questionnaire for evaluation of satisfaction of the course (1 point, strongly disagree; 2 points, disagree; 3 points, neither; 4 points, agree; 5 points, strongly agree).

## Course Structure

### Theoretical Lectures

The course started with a 3-day lecture series aimed to take the residents through the microsurgical process of planning and execution. The initial day covered the foundations of reconstructive theory, flap classifications, terminology, and microvascular anatomy. Day 2 delved into operative planning of flap inset, pedicle position, and postop monitoring. On the third day, attendees were introduced to flap harvesting, aided by detailed surgical step-by-step videos and valuable surgical pointers.

### Hands-on Training

**Fig. 2:**
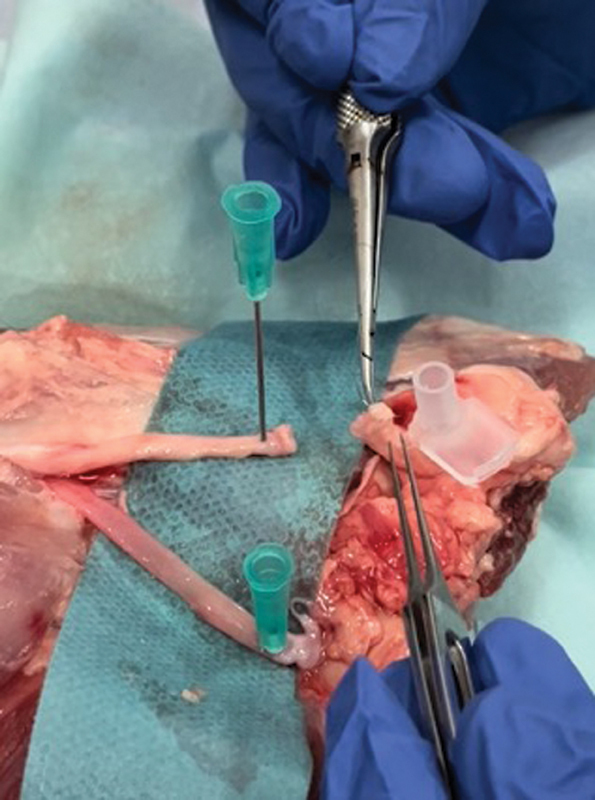
A student performing an anastomosis using 7/0 nylon.

On the fourth day, practical surgical training took the center stage. Residents, grouped in pairs, practiced vascular and nerve anastomosis on porcine thigh models, under the guidance of instructional videos. They were equipped with basic microsurgical instruments and had a 5-hour window to complete their tasks ( Fig. 2 ). Real-time guidance was available through a looped video tutorial, and the session concluded with a patency check of the anastomoses.

## Results

All participants successfully executed an average of three anastomoses each, which speaks volumes about the efficacy of the course and the chosen model. The postcourse feedback highlighted the structured approach of the course, with both lectures and practical elements as its strongest aspect ( Table 2 ). Despite varying levels of prior experience, the porcine model aided all in completing their tasks and all anastomoses were found patent. The feedback was overwhelmingly positive, with the majority affirming the course relevance, utility, and impact on their skills and future aspirations. Feedback further revealed that working collaboratively was beneficial. Most were satisfied with their contributions, and while a significant number expressed a desire to attend an international course, the majority were content with the local setup. Participants largely agreed on the adequacy of time and instruments provided, with some recommendations for future courses, such as extended durations, increased practical training, and follow-up sessions.

**Table 2:** The results from the 18-question 5-point Likert scale questionnaire

Question number	Strongly disagree (1)	Disagree (2)	Neither (3)	Agree (4)	Strongly disagree (5)
**1**	0	0	0	0	17 (100%)
**2**	0	0	0	0	17 (100%)
**3**	0	0	0	2 (11.8%)	15 (88.2%)
**4**	0	0	0	0	17 (100%)
**5**	0	0	0	0	17 (100%)
**6**	0	0	0	0	17 (100%)
**7**	0	0	0	0	17 (100%)
**8**	0	0	0	0	17 (100%)
**9**	0	0	0	0	17 (100%)
**10**	0	0	0	0	17 (100%)
**11**	0	0	0	0	17 (100%)
**12**	0	0	0	2 (11.8%)	15 (88.2%)
**13**	0	0	7 (41.2%)	2 (11.8%)	8 (47.1%)
**14**	0	0	1 (5.9%)	2 (11.8%)	14 (82.4%)
**15**	0	0	1 (5.9%)	5 (29.4%)	11 (64.7%)
**16**	0	0	0	0	17 (100%)
**17**	0	0	0	0	17 (100%)
**18**	0	0	0	0	17 (100%)

## Discussion

Training in reconstructive microsurgery is a challenging endeavor that requires an appropriate educational environment, tools, and sequence in complexity progression. Animal models arguably simulate human conditions the closest, and can thereof provide the optimal vascular simulation model. This study provides an insight into the feasibility of successful vascular anastomotic training in the porcine thigh by junior residents as a first step in the skills progression from large to small diameter vascular anastomotic training.

An optimal starting point for novice trainees in their journey of reconstructive surgical practice is training on simple to complex models, 2 and a complete animal-based pathway has been shown to be preferred by residents. 3 The porcine model is particularly advantageous for beginners, but has only been described in three papers thus far. 1 4 5 Despite the large-diameter structures in the porcine thigh not providing a true microsurgical environment, adopting a correct suturing technique in initially larger structures could improve speed and efficacy in the later performance on true microsurgical anastomoses. 1 Encouraging novice residents to embrace the challenges posed by microsurgery is essential, and in this regard, the porcine thigh model serves not just as a stepping stone but as a catalyst; inspiring aspiring surgeons to delve in to the vast, challenging, and rewarding realm of microsurgery. Furthermore, plastic surgery residents should arguably seek to develop their training further by gaining a deeper understanding of reconstructive theory through lectures as well. 6 Collaborating with international units, both in training courses and surgically, can further improve knowledge, skills, and patient outcomes. Medical educationalists and specialized medical training centers play a vital role in facilitating this learning process, and can form an important part of training courses. Structured training programs, workshops, and mentorship are indispensable in shaping future microsurgeons. The balanced amalgamation of theoretical knowledge, practical skills, and ethical considerations is integral in molding competent, empathetic, and innovative practitioners.

In conclusion, the meticulous introduction of novice residents into the world of microsurgery is imperative for fostering understanding of its interdisciplinary role, complexity, and precision in reconstructive surgery. The utilization of the porcine thigh model is optimal for providing realistic, hands-on experience to junior trainees and can serve as a suitable stepping stone in a fully animal-based large-to-small caliber vascular skills progression. It is this dedication to learning and mastery of skills, combined with a passion for innovation and a commitment to patient care, that will continue to drive the advancements in reconstructive microsurgery, pushing the boundaries of what is medically possible. Furthermore, active collaboration between plastic surgery units is vital for sharing knowledge and improving plastic surgery training, microsurgical practice and patient outcomes worldwide.
